# Identification of Epigenetic-Dysregulated lncRNAs Signature in Osteosarcoma by Multi-Omics Data Analysis

**DOI:** 10.3389/fmed.2022.892593

**Published:** 2022-06-16

**Authors:** Junchao Huang, Jingwei Zhang, Haijun Xiao

**Affiliations:** Department of Orthopedics, Anhui University of Science and Technology Affiliated Fengxian Hospital, Shanghai, China

**Keywords:** osteosarcoma, histone modification, long non-coding RNAs, multiomics, prognosis

## Abstract

**Background:**

Alterations of epigenetic modification patterns are potential markers of cancer. The current study characterized six histone modifications in osteosarcoma and identified epigenetically dysregulated long non-coding RNAs (epi-lncRNAs).

**Methods:**

Multi-omics data were obtained from osteosarcoma cell line SJSA1 and a normal cell line. Differentially expressed lncRNAs (DElncRNAs) between osteosarcoma and normal skeletal muscle were analyzed using Limma. MACS2 was applied to identify the “peaks” modified by each histone in the cell. Promoters or enhancers of DElncRNA were overlapped with differential histone-modified regions (DHMR) to screen epi-lncRNAs. Univariate and multivariate Cox regression analysis were performed to detect the genes closely related to the prognosis of osteosarcoma and to construct risk models.

**Results:**

A total of 17 symbolic epi-lncRNA in osteosarcoma were screened, and 13 of them were differentially expressed between osteosarcoma and normal samples. Eight epi-lncRNAs were retained by Univariate Cox regression analysis. Four of these epi-lncRNAs were used to construct an epi-lncRNA signature. The risk score of each osteosarcoma sample in the high- or low-risk group was estimated according to the epi-lncRNA signature. The overall survival (OS) of the low-risk group was significantly better than that of the high-risk group. The area under the receiver operating characteristic (ROC) curve of the model was 0.79 and 0.82 for 1-, 3-, and 5-year OS, respectively.

**Conclusion:**

Our results revealed the histone modification pattern in osteosarcoma and developed 4-epi-lncRNA signature to predict the prognosis of osteosarcoma, laying a foundation for the identification of highly specific epigenetic biomarkers for osteosarcoma.

## Introduction

Osteosarcoma is the most common primary malignant tumor in children and adolescents (1). This tumor mainly occurs in the long bones (femur, tibia, humerus), the growth plates near the diaphysis, as well as all areas characterized by extensive bone rearrangement, and occurs less frequently in flat bones and the spine (2). Osteosarcoma is characterized by the presence of transformed osteoblastic cells producing osteoid matrix. Currently identified subtypes of osteosarcoma include typical intramedullary or central (osteoblastic, chondroblastic and fibroblastic); telangiectasic; small cell; high-grade surface; secondary osteosarcoma; parosteal; periostal; and central with a low degree of malignancy (3). Osteosarcoma exhibits a high tendency for local invasion and metastasis, and although many factors that predict metastasis have been identified, there is no effective therapeutic strategy other than surgery to reduce the number of patients with metastatic disease or to cure these patients with metastatic disease (4). At advanced stages, the survival rate of these metastatic patients is still only 20% (5). Therefore, improving the therapeutic unit of osteosarcoma there remains a constant and primary goal for many global research and clinical communities.

Epigenetics is the study of alterations caused by modifications in gene expression patterns that occurred during organismal development or cell proliferation without any change in DNA sequences (6). Histone modification could be resulted from the regulation of chromatin condensation level, and is therefore important in regulating gene expression and other nuclear events. Histone modification together with DNA methylation constitute are the foundation of epigenetic regulation of cell functions (7). Epigenetic modifications that work in conjunction with genetic mechanisms, which regulate transcriptional activity, are maladjusted in many diseases, including in cancers (8). Several epigenetic drugs, including inhibitors of EZH2, IDH, histone deacetylases (HDACis), and DNA methyltransferases (DNMTs) with many others undergoing clinical trials for treating solid have been designed to reverse cancer-specific epigenetic modification to normal epigenetic state, and have been approved by Food and Drug Administration (FDA) (9). To date, the efficacy and use of epigenetic therapy have been demonstrated mainly in the treatment of hematological malignancies, with limited supporting data for solid malignancies (10). Epigenetic therapies also face problems with their lack of specificity for cancer cells. Targeting a combination of epigenetic modifications specific to or preferentially present in cancer cells is a feasible strategy (11).

Several previous studies have shown that lncRNA transcription is affected by epigenetic mechanisms, including DNA methylation and histone modification. Abnormally low level of DNA methylation mediates the activation of lncRNA SNHG12, enabling it to play a role in glioblastoma resistance (12). Analysis on the relationship between lncRNA expression and histone modification of its promoter showed that trimethylation of histone H3 at lysine 4 (H3K4me3) and histone H3 trimethylated at lysine 27(H3K27me3) are generally correlated to lncRNA activation and repression, respectively (13). A study integrating bioinformatics with *in vitro* and *in vivo* biological experiments demonstrated that HOXC-AS3 is obviously activated by gain of H3K4me3, and that H3K27ac is involved in the regulation of gastric cancer (14). In basal-like breast cancer, the expression of BLAT1 is regulated at epigenetic level by DNA methylation of the CpG island in the promoter. On the contrary, lncRNAs also mediate epigenetic modification. Mohammad reported a lncRNA that can induce DNA methylation in specific regions of the Kcnq1 locus (15). Endogenous unspliced lncRNA ANRASSF1 binds to its transcriptional site to form an RNA/DNA hybrid, which then binds to polycomb repressive complex 2. This complex is only recruited to the RASSF1A promoter and could increase H3K27me3 repressive histone mark (16). The relationship between lncRNAs and epigenetic modifications may be exploited as an effective regulator of epigenetic mechanism (17). However, the data collected so far on the involvement of lncRNA in epigenetic regulation is still the tip of the iceberg in this emerging field, and the further expansion of these data may simultaneously reveal a large number of undeveloped targets and pathways suitable for epigenetic therapy (18).

**Figure 1:**
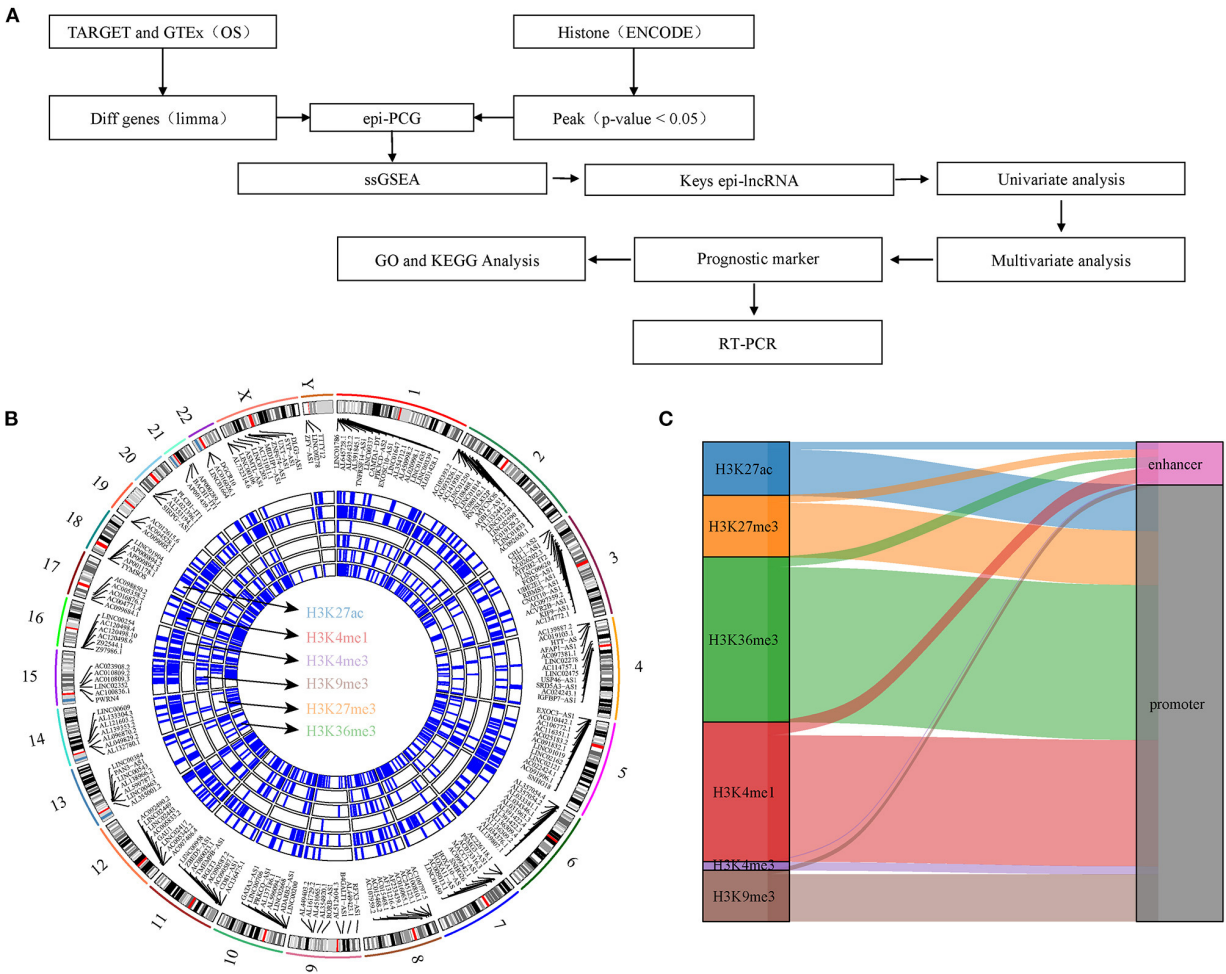
Work flow chart and genomic landscape of epi-lncRNAs. **(A)** Workflow diagram of the study. **(B)** The locations on the chromosome of epi-lncRNAs shows the global apparent modification distribution. Epi-lncRNAs is marked in the appropriate location. **(C)** Alluvial diagram of six histone modification distributions of promoter and enhancer.

In this study, we explored the relationship between histone modification and abnormal lncRNA expression through integrating six histone modified CHIP-seq data and RNA-seq data of osteosarcoma. We analyzed epigenetic-dysregulated lncRNAs (epi-lncRNAs) and its genome landscape, and identified osteosarcoma-specific epi-lncRNAs to develop a prognostic signature, which may be of great significance to improve the understanding of the tandem of lncRNAs and histone modification. Figure 1A shows the overall design process of this study.

## Materials and Methods

Six groups of histone modified replicated narrowPeak data were acquired from osteosarcoma cell line SJSA1 in Encyclopedia of DNA Elements (ENCODE) (19), including histone H3 monomethylated at lysine 4 (H3K4me1), H3K4me3, histone H3 trimethylated at lysine 9 (H3K9me3), histone H3 acetylated at lysine 27(H3K27ac), H3K27me3, histone H3 trimethylated at lysine 36 (H3K36me3). Normal human osteoblast was used as a control. Transcriptome data of osteosarcoma came from TRAGET database (https://ocg.cancer.gov/programs/target). The transcriptome data of normal skeletal muscle were obtained from GTEx database (https://www.genome.gov/Funded-Programs-Projects/Genotype-Tissue-Expression-Project). The batch effect of the two datasets was eliminated by removeBatchEffect in LIMMA R package. The GTF file (version 40, https://ftp.ebi.ac.uk/pub/databases/gencode/Gencode_human/release_40/gencode.v40.annotation.gff3.gz) of the transcript was obtained from GENCODE database (https://www.gencodegenes.org/human/), and the lncRNA and protein coding genes were determined according to the geneType attribute.

### Identification of Epigenetic-Dysregulated lncRNAs and Protein-Coding Genes

The difference between osteosarcoma and normal skeletal muscle was analyzed by Limma (20) to obtain differentially expressed lncRNA (DElncRNAs) and PCGs (DEPCGs). The threshold was adjusted by benjamini-Hochberg method (*P* < 0.05). Histone-modified “peaks” with a region of *q* < 0.05 in each cell line of the six groups was detected by MACS2 (https://github.com/taoliu/MACS/) (21). The upstream 2 kb and downstream 0.5 kb of the transcriptional initiation site (TSS) were defined as promoters, which were identified by CHIPseeker (22). The chromatin immunoprecipitation sequencing (ChIP-seq) data of H3K27ac in FANTOM5 were integrated into our study, and high H3K27ac signal was defined as an active enhancer. Differential histone modified regions (DHMR) were screened by MACS2 bdgdiff. DElncRNAs/DEPCGs with at least one promoter or enhancer overlapping with DHMR were identified as epigenetic-dysregulated lncRNAs/PCGs (epi-lncRNAs/epi-PCGs).

### Identification of Genomic Characteristic for Epigenetic Modifications

To examine the genomic characteristics of epigenetic disorders of lncRNA/PCGs, genes were divided into four different groups, namely, epi-lncRNAs, non-epi-lncRNAs, epi-PCGs, non-epi-PCGs, according to the characteristics and gene types of modification. The number and length of exons and transcripts in each group were summarized, and differences between epi-lncRNAs/epi-PCGs and non-epi-lncRNAs/non-epi-PCGs were compared. In addition, different genomic distributions of abnormally epigenetically modified lncRNA were analyzed.

### Single Sample Gene Set Enrichment Analysis

Lnc2Cancer is a database providing lncRNA-cancer correlations between 216 human cancers subtypes and 2,659 lncRNAs (23). The relationship between epi-lncRNAs and different types of cancer was analyzed using Lnc2Cancer database, and epi-lncRNAs related to osteosarcoma were screened. The ssGSEA scores of osteosarcoma samples were evaluated by R packet “GSVA” (24). At the same time, the Kyoto Encyclopedia of Genes and Genomes (KEGG) pathway score of each sample was calculated to investigate the relationship between osteosarcoma-related epilncRNAs and different biological pathways.

### Construction of Osteosarcoma Prognosis-Related Signature Based on epi-lncRNAs

Univariate Cox regression analysis on osteosarcoma-related epi-lncRNAs and overall survival (OS) was performed to screen OS-related epi-lncRNAs in osteosarcoma. Furthermore, based on its expression, max_stat R package was used to evaluate the risk of osteosarcoma samples. Then stapAIC with step-by-step regression was used to eliminate unnecessary epi-lncRNAs, and then multivariate Cox regression analysis was conducted to identify closely related genes and build an epi-lncRNAs risk model with the strongest prognostic significance.

### Functional Enrichment Analysis on the epi-lncRNAs Risk Model

The Pearson correlation analysis was performed to analyze the correlation between epi-PCGs and epi-lncRNA in TARGET (threshold: | Corr | > 0.4 epi-PCGs *P* < 0.05). The epi-PCGs identified were integrated into the “WebGestaltR” R package (25) for KEGG and Gene Ontology (GO) analysis to further evaluate its functional characteristics. FDR < 0.05 was considered as the pathway of significant enrichment.

### Cell Culture

Human SV40-transfected osteoblasts hFOB1.19 (BNCC255176), human osteosarcoma cell line U2OS (item number) and 143B (BNCC337683) were obtained from BeNa Culture Collection (Beijing, China). All of them were subcultured in dulbecco's modified eagle medium (DMEM) containing 10% fetal bovine serum and 1% penicillin-streptomycin at 37°C and 5% CO_2_ concentration.

### Quantitative Real-Time PCR

The cells in logarithmic growth phase were taken and their density was adjusted to 1 × 10^5^ cells/mL and then inoculated into 6-well cell culture plates with 2 mL per well and 3 multiple holes in each group. TRIzol reagents (Invitrogen, Carlsbad, California, USA) were used to extract total RNA. We followed the instructions provided by the manufacturer. Then, RevertAid First Strand cDNA Synthesis kit (Thermo Fisher Scientific, United States) was used for reverse transcription of RNA to synthesize cDNA. qRT-PCR was performed on the ABI StepOnePlus Real-Time PCR System (Applied Biosystems, Foster City, CA, USA) by utilizing SYBR Green qPCR Master Mix (2x) (Bimake, United States) and 40 cycles were amplified. All expression data was normalized to β-actin as an internal control utilizing the 2–ΔΔCt method. The primer sequences involved are as follows:

A2M-AS1 – forward: AGCCTACTCAGACCGACA

A2M-AS1- reverse: GAAATGCTTGAAGACCAC

CACNA1G-AS1-forward: GGACAGAAGACACCAAGGG

CACNA1G-AS1-reverse: GAGTTGCGAAGGCAGTTA

LBX2-AS1-forward: TAGAAGCCGTGGAGTCAG

LBX2-AS1-reverse: TTCAAGGAACAAAAGGGA

NNT-AS1-forward: GACTGCTTTGAGGATTTG

NNT-AS1-reverse: GAGTGACATTCTTTACTACCG

β-actin-froward: AGCGAGCATCCCCCAAAGTT

β-actin -reverse: GGGCACGAAGGCTCATCATT.

### Statistical Analysis

All statistical data were analyzed and the results were presented by R language v4.0.2 (https://www.r-project.org/). The Kaplan-Meier method and log-rank Tests were used to compare the sample OS. The accuracy of the epi-lncRNAs risk model was evaluated by drawing the Receiver Operating Characteristic (ROC) curve. *P* < 0.05 was defined as having a statistical significance standard.

## Results

### Identification of epi-lncRNAs and epi-PCGs

In this study, DHMR was identified by CHIP-seq analysis on six histone-modified markers. A total of 9,995 lncRNAs and 5,141 PCGs were screened from osteosarcoma by difference analysis. 1,148 epi-lncRNAs, 12,902 non-epi-lncRNAs, 1,729 epi-PCGs and 17,805 non-epiPCGs were identified by overlapping analysis on DElncRNA/DEPCG promoter or enhancers with DHMR. The number of transcripts and exons in epi-lncRNAs was significantly higher than that in non-epi-lncRNAs. The exon of epi-lncRNAs was also significantly longer than that of non-epi-lncRNAs. The number of transcripts and exons of Epi-PCG was significantly fewer than that of non-epi-PCGs (Supplementary Figure S1). The locations of abnormal histone-modified epi-lncRNAs on the chromosome showed a wide distribution of apparent modification, noticeably, H3K4me1, H3K9me3, H3K27ac, H3K27me3, and H3K36me3 were the main histone-modified epi-lncRNAs (Figure 1B). These histone modifications are mainly covered in the promoter region (Figure 1C).

### Biological Characteristics of Histone Modification Regulation

**Figure 2:**
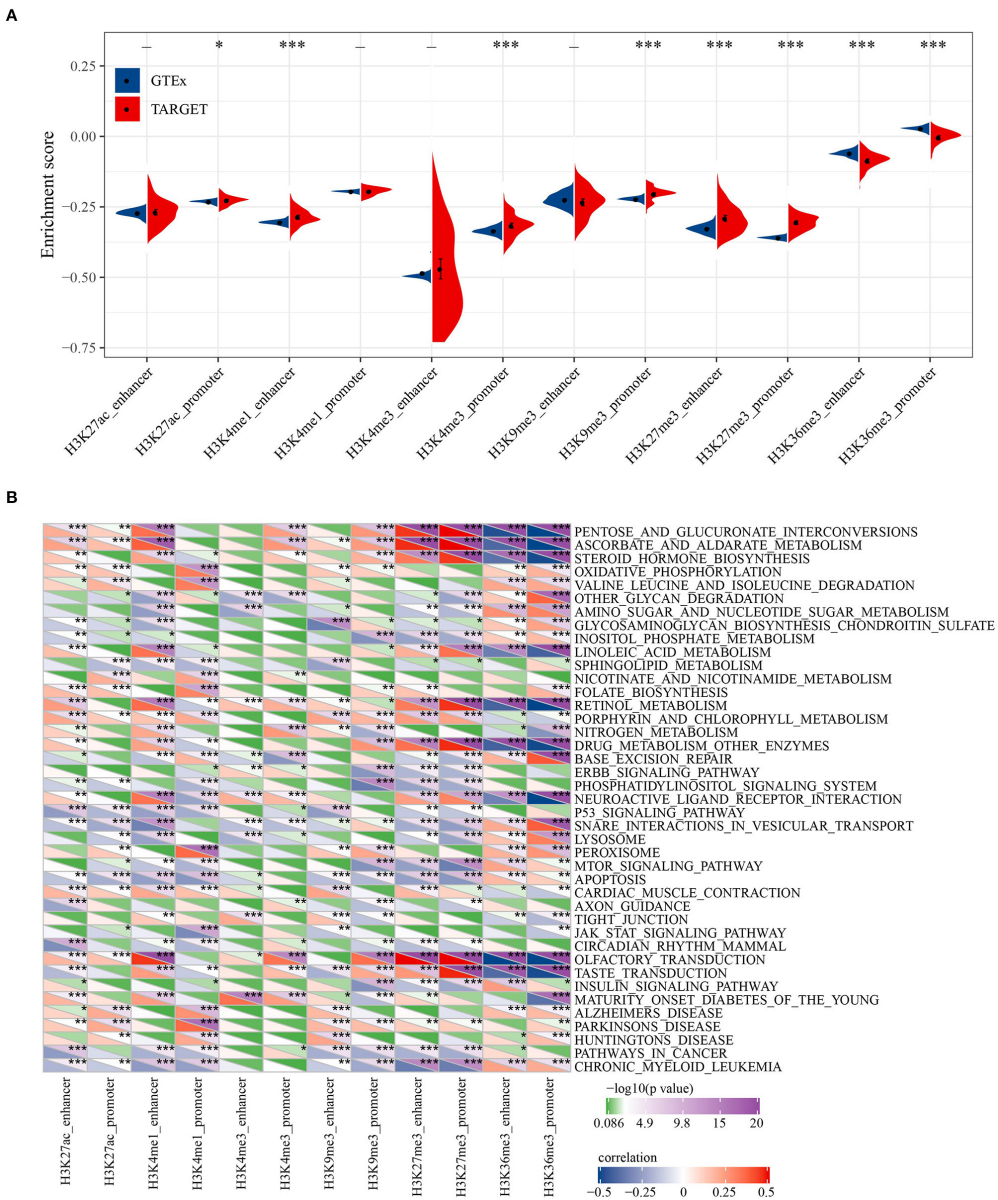
Biological characteristics of histone modification regulation. **(A)** The difference for enrichment scores of six histone modified promoters and enhancers between normal samples and osteosarcoma samples. **(B)** Correlation between KEGG pathway and six histone-modified promoters and enhancers. *P < 0.05, **P < 0.01, ***P < 0.001.

To reveal the biological significance of lncRNA regulated by epigenetic dysregulation, ssGSEA was performed to calculate the score of each osteosarcoma sample. The enrichment scores of six histone-modified promoters and enhancers in normal samples and osteosarcoma samples were determined. Significant differences were found in H3K27ac promoter, H3K4me1 enhancer, H3K4me3 promoter, H3K9me3 promoter, H3K27me3 enhancer, H3K27me3 promoter, H3K36me3 enhancer, and H3K36me3 promoter between normal samples and osteosarcoma samples. Osteosarcoma samples showed high enrichment scores of H3K27ac promoter, H3K4me1 enhancer, H3K4me3 promoter, H3K9me3 promoter, H3K27me3 enhancer and H3K27me3 promoter, but low enrichment scores of H3K36me3 enhancer and H3K36me3 promoter (Figure 2A). Pearson correlation analysis showed that 41 KEGG pathways were related to most of the six histone-modified promoters and enhancers. The KEGG pathways involved in malignant tumors included metabolism, cancer cell proliferation, apoptosis, autophagy, etc. (Figure 2B).

### Identification of epi-lncRNAs Specifically Expressed in Osteosarcoma

**Figure 3:**
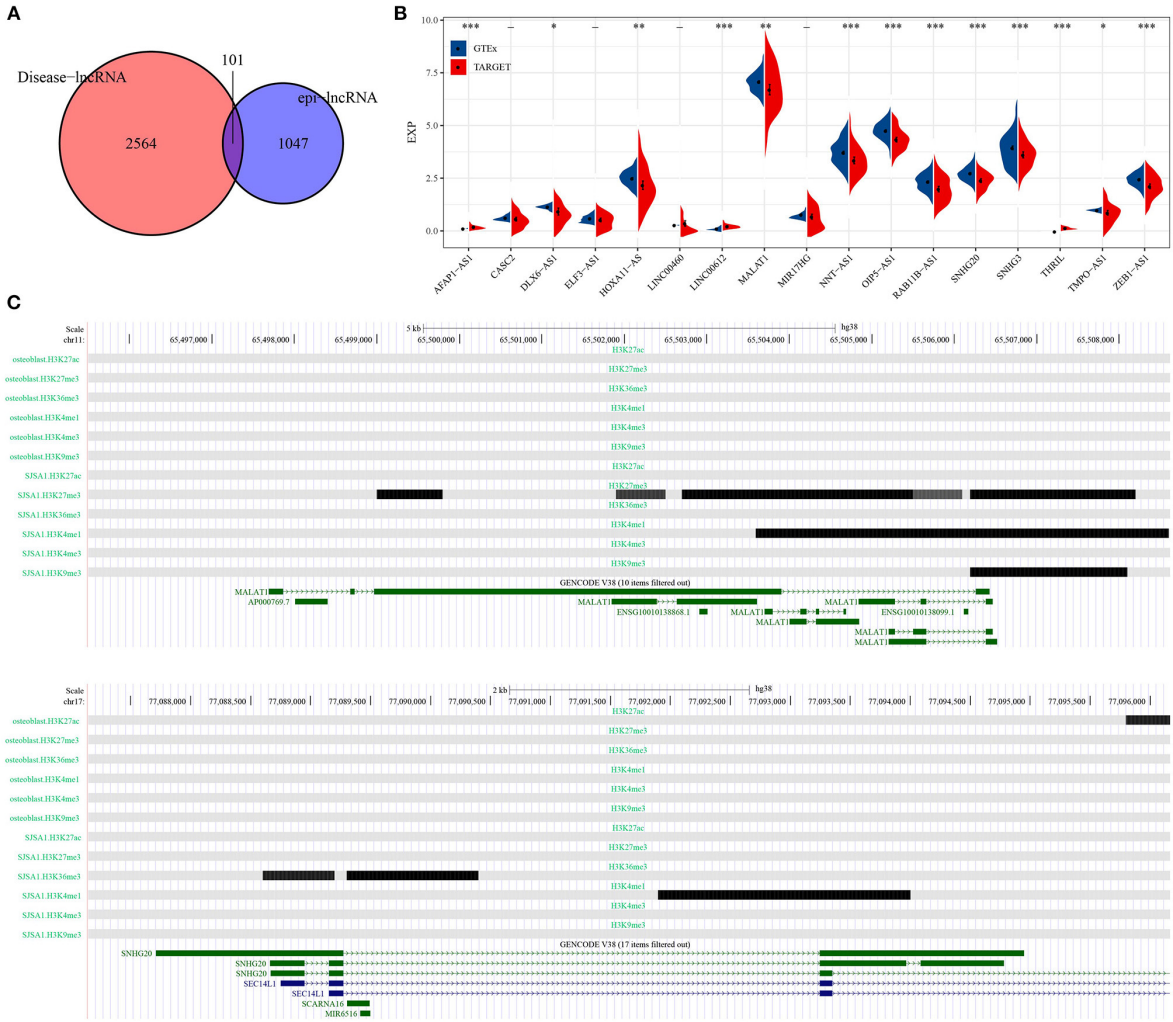
Identification of epi-lncRNAs specifically expressed in osteosarcoma. **(A)** The venn diagram of the intersection of cancer-related lncRNAs and osteosarcoma epi-lncRNAs in Lnc2Cancer. **(B)** Analysis of the expression of 17 osteosarcoma emblematical epi-lncRNAs in normal samples and osteosarcoma samples. **(C)** Histone modification profiles of MALAT and SNHG20. *P < 0.05, **P < 0.01, ***P < 0.001.

A total of 101 osteosarcoma-related epi-lncRNAs were found in epi-lncRNAs through Lnc2Cancer database (Figure 3A), and 17 epi-lncRNAs were selected as the markers for osteosarcoma. Through the difference analysis on 17 epi-lncRNAs between normal samples and osteosarcoma samples, 13 DEepi-lncRNAs, including 10 abnormally down-regulated epi-lncRNAs and 3 abnormally down-regulated epi-lncRNAs, were obtained (Figure 3B). MALAT showed increased H3K27me3 enrichment in promoter and distal enhancer in SJSA1, while the downstream region of MALAT was significantly enriched by H3K4me1 and H3K9me3. On the other hand, the H3K36me3 enrichment of SNHG20 promoter in SJSA1 increases, and the downstream region was covered by H3K4me1. It should be noted that H3K4me1 and H3K36me3 were enhancer markers, while H3K27me3 and H3K9me3 were inhibitory markers, indicating that a variety of histone modifications reshaped the acquisition/loss of active promoters and/or enhancers through enrichment at different sites of genes, which could synergistically affect gene expression (Figure 3C).

### Construction of an Osteosarcoma Prognostic Signature Based on epi-lncRNAs

**Figure 4:**
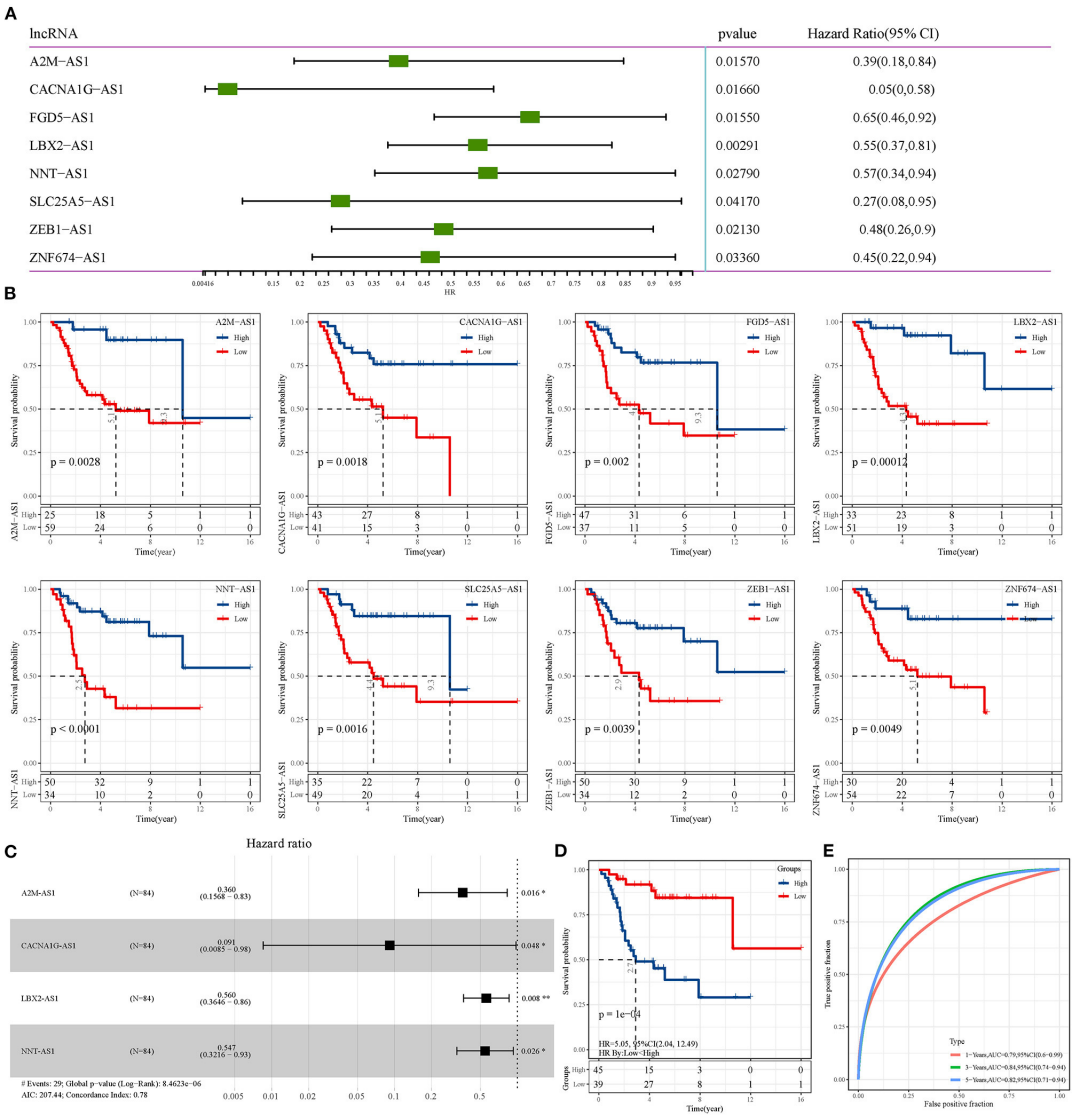
Construction of osteosarcoma prognostic signature based on epi-lncRNAs. **(A)** Univariate Cox regression analysis of 8 epi-lncRNAs significantly correlated with OS of osteosarcoma. **(B)** The OS of osteosarcoma patients was evaluated according to the expression of each of the 8 epi-lncRNAs. **(C)** Forest map of 4 epi-lncRNAs by multivariate Cox regression analysis. **(D)** The survival curve of patients with different risks assessed by the 4-epi-lncRNA signature. **(E)** The ROC curve shows the area under curve (AUC) of the 1-, 3-, and 5-year OS calculated by the 4-epi-lncRNA signature.

Univariate Cox regression analysis of epi-lncRNAs related to osteosarcoma identified 8 epi-lncRNAs significantly correlated with OS of osteosarcoma (Figure 4A). Each OS-related epi-lncRNA can group osteosarcoma samples according to its expression, and the survival of different risk groups can be clearly distinguished (Figure 4B). To integrate establish an overall effective osteosarcoma scoring model, 4 epi-lncRNAs were removed according to stepAIC, and the remaining four epi-lncRNAs (A2M-AS1, CACNA1G-AS1, LBA2-AS1, and NNT-AS1) were recruited to build a risk model (Figure 4C). According to the 4-epi-lncRNA signature, the samples were divided into two risk groups with significant OS differences. Specifically, the OS of the low-risk group was much better than that of the high-risk group (Figure 4D). The area under ROC curve of 4- epi-lncRNA signature reached 0.79, 0.84, and 0.82 in 1-, 3-, and 5-year OS, respectively (Figure 4E), indicating a high prediction accuracy. In addition, we also evaluated the relationship between these lncRNAs and immunity. It can be observed that LBX2-AS1 has a significant positive correlation with a variety of immune infiltrating cells, such as Activated dendritic cell, Macrophage and Central memory CD8 T cell, and CACNA1G-AS1 has a significant negative correlation with a variety of immune infiltrating cells, such as Type 17 T helper cell, Immature dendritic cell and Activated dendritic cell (Supplementary Figure 2A). Further, we also observed that patients in the high-risk group have significantly higher Activated CD8 T cell infiltration (Supplementary Figure 2B) and lower fibroblasts and CD8 T cells infiltration (Supplementary Figure 2C), Similarly, the matrix score in high-risk patients was significantly lower than that in low-risk patients (Supplementary Figure 2D).

### Expression of lncRNA in 4-epi-lncRNA Signature

**Figure 5:**
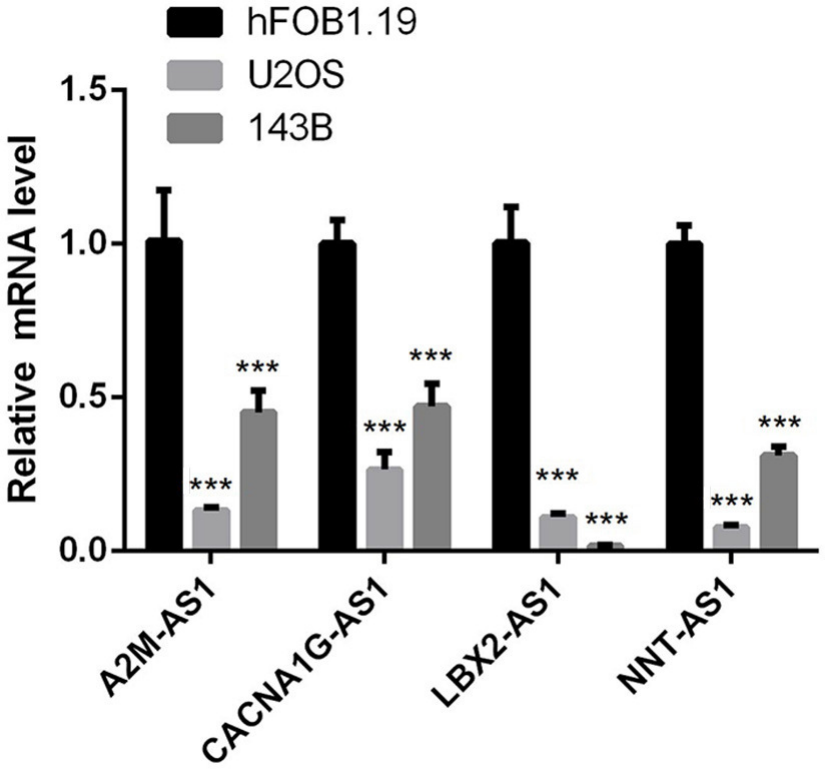
qRT-PCR was used to detect the expression of lncRNA in human osteoblast and osteosarcoma cells in 4-epi-lncRNA signature. (*P < 0.05, ** P < 0.01, *** P < 0.001, **** P < 0.0001).

To verify whether lncRNAs in the 4-epi-lncRNA signature are abnormally expressed in osteosarcoma as predicted, we detected their expression levels in human osteoblasts and osteosarcoma cells by qRT-PCR. The results showed that the expression of A2M-AS1, CACNA1G-AS1, LBA2-AS1, and NNT-AS1 was significantly lower in osteosarcoma cells U2OS and 143B compared with hFOB1.19 cells (Figure 5). According to the results of survival analysis obtained by Figure 4B, the low expression of these four lncRNA was associated with poor prognosis. This also implies to some extent that our predicted trend is of practical significance.

### Regulatory Role of epi-lncRNAs in Prognosis of Osteosarcoma

**Figure 6:**
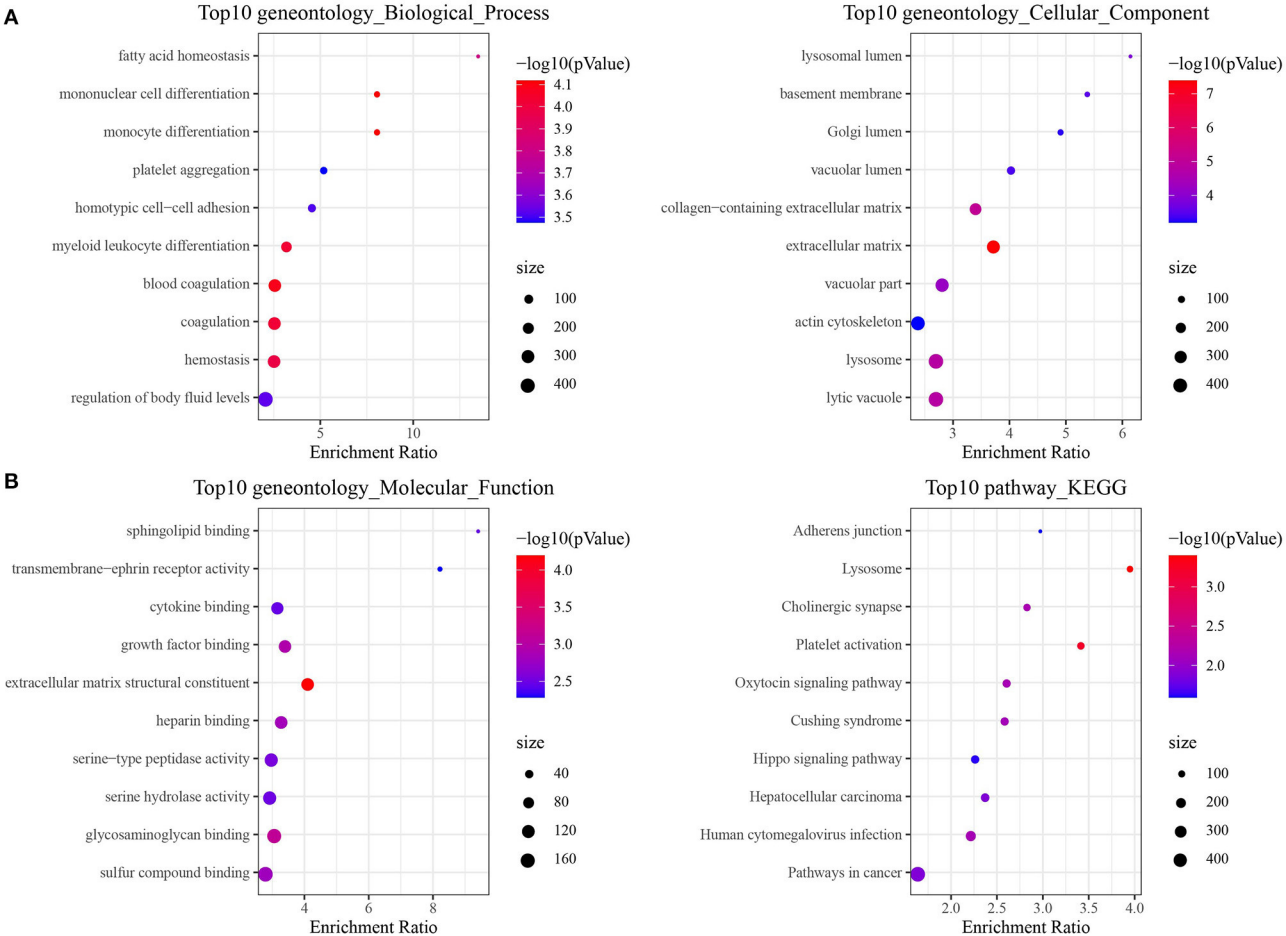
Enrichment pathway analysis of epi-lncRNAs in osteosarcoma prognostic signature. **(A)** GO analysis of 486 epi-lncRNAs-related PCGs, including biological process (BP), cellular component (CC) and molecular function (MF). **(B)** KEGG analysis of 486 epi-lncRNAs-related PCGs showed the highest 10 pathways of enrichment ratio.

LncRNAs play a critical role in cancer through directly or indirectly regulating the coding genes. Therefore, the function of epi-lncRNAs-related PCGs in prognostic signature was studied. Firstly, 486 PCGs related to epi-lncRNAs in TARGET were filtered by Pearson correlation analysis (306 PCGs with positive correlation, 180 PCGs with negative correlation). GO analysis based on these PCGs demonstrated that the most relevant GO biological process (BP) included fatty acid homeostasis, mononuclear cell differentiation, homotypic cell–cell adhesion and myeloid leukocyte differentiation; the most related cellular component (CC) were lysosomal lumen, basement membrane and Golgi lumen and vacuolar lumen. Most of the PCGs were enriched in the signal molecules binding (cytokine binding, growth factor binding) and cellular activity-related molecules in TME (Figure 6A). KEGG analysis based on 486 PCGs showed that the PCGs were enriched in a variety of pathways, which regulate cancer, such as adherens junction, Hippo signaling pathway, hepatocellular carcinoma, and pathways in cancer (Figure 6B). In addition, we also analyzed the expression of genes downstream of the four key lncRNAs. It can be observed that TLX2 and PAIP1 in the latest four genes downstream of the four lncRNAs are significantly related to poor prognosis, suggesting that these genes may affect the expression of downstream genes through cis-regulation (Supplementary Figure 3A). Further, the targeted miRNAs of the four lncrnas are predicted from the microT, miRanda, mircode, miRDB, miRmap, miRtarbase, PicTar, PITA, RNA22, starbase, TargetMiner and TargetScan database according to the Cerna hypothesis. Then, we use 12 databases to predict the target encoding gene (mRNA) of miRNA and retain the target mRNA that appears in at least 6 databases. The ceRNA network is constructed. The network contains 4 lncRNA, 14 miRNA and 325 mRNA (Supplementary Figure 3B). Using the gene analysis of the regulatory network of the ceRNA, we can observe that these genes are enriched into many tumor pathways, such as liver cancer, breast cancer, Glioma and non-small cell lung cancer etc. (Supplementary Figure 3C). These results show that the potential ceRNA networks of these four lncRNAs are closely related to the occurrence and development of tumors.

## Discussion

Histone modification, a common epigenetic mechanism, is the process of histone modification by enzymes, including post-translational modifications such as methylation, acetylation, phosphorylation and ubiquitin (26). Recent evidence suggests that post-translational modification of histones accompanied by lncRNAs profiles is involved in several clinical cancer parameters, including histopathology progression, prognosis, and/or responsiveness to unique or combined oncological therapies (27). In this work, we explored the relationship between histone modification and abnormal lncRNAs in osteosarcoma according to 6 emerging classes of histone modifications subjected for epigenome profiling by International Human Epigenome Consortium (http://www.ihec-epigenomes.org/), and screened epi-lncRNAs in osteosarcoma.

The difference of RNA modification pattern between normal tissue and osteosarcoma tissue was explored, and we found that osteosarcoma samples showed high levels of H3K27ac promoter, H3K4me1 enhancer, H3K4me3 promoter, H3K9me3 promoter, H3K27me3 promoter and enhancer enrichment scores, but low levels of H3K36me3 promoter and enhancer enrichment scores, indicating the diversity of histone modification in osteosarcoma. These combinations of epigenetic modifications that stimulate cancer cell development are considered to be candidate targets for cancer therapy (28). In osteosarcoma, we found 13 specific lncRNAs related to these histone modifications, and analyzed histone modification spectra of two of them. The results demonstrated that MALAT showed increased H3K27me3 enrichment in promoter and distal enhancer in osteosarcoma cell lines, while the downstream region was significantly enriched by H3K4me1 and H3K9me3. On the other hand, the H3K36me3 enrichment of SNHG20 promoter in SJSA1 increased, and the downstream region was covered by H3K4me1. These two kinds of lncRNAs were found to be greatly overexpressed in osteosarcoma cells (29, 30). The modification activity of these active histones may be an important factor in regulating their expression.

Finally, 4 epi-lncRNAs were identified from 13 epi-lncRNAs specifically expressed in osteosarcoma to develop a prognostic signature for survival prediction of osteosarcoma. The function of A2M-AS1 as a positive regulatory factor to promote the metastasis of breast cancer has been characterized, and is related to a poor prognosis (31). CACNA1G-AS1 is highly expressed in colorectal cancer (32) and hepatocellular carcinoma tissues (33), which accelerates the malignant biological process of both cancers. The up-regulated expression of LBX2-AS1 in gastric cancer cells and tissues contributes to the malignant transformation of this cancer (34). In ovarian cancer, LBX2-AS1 is also an oncogenic factor, enhancing the proliferation and migration of cancer cells and promoting the formation of solid tumors (35). NNT-AS1 induces cell proliferation and invasion in prostate cancer (36) and lung cancer (37). Notably, herein, qRT-PCR was utilized to detect the expression of these four lncRNAs in human osteoblasts and osteosarcoma cells, and we found that compared with human osteoblasts, the expression of all of this lncRNA in osteosarcoma cells was significantly down-regulated. Survival analysis showed that low expression of each of the four lncRNAs was significantly associated with a good prognosis in patients with osteosarcoma. Therefore, our results suggested that all 4 lncRNAs are protective factors in osteosarcoma. We speculated that the reason for this result might be the heterogeneity of lncRNA between tumors. At present, the relationship between them and histone modification is still unclear. LncRNAs located in the nucleus are involved in chromatin interactions, transcriptional regulation and RNA processing, while cytoplasmic lncRNAs can modulate mRNA stability or translation and influence cellular signaling cascades (38). It can be seen that lncRNA regulates cell behavior by affecting PCG. Therefore, we finally analyzed four epi-lncRNA-related PCGs and analyzed the functions of these PCGs. There are various pathways enriched by PCGs, and the most notable ones were adherens junction, Hippo signaling pathway, hepatocellular carcinoma and pathways in cancer. Adherens junction is the initiator and maintenance of adhesion between cancer cells and regulation of tumor cell proliferation and migration (39). Hippo signaling pathway was identified by Atlas as one of the eight major signaling pathways in human cancers (40). Therefore, we speculated that the 4 epi-lncRNAs we identified may interact with the screened PCGs through specific mechanisms and ultimately regulate the pathological progression of osteosarcoma.

Our research has certain potential limitations. Firstly, DNA methylation is the most important modification in epigenetics, we did not analyze the effects of DNA methylation. Secondly, Innovations in bioinformatics analysis are not abundant, and the limited sample size prevented us from addressing specific epigenetic or transcriptome differences between potentially important epigenetic modification factors such as age. Finally, Our data are all from public data sets, and the functional and molecular mechanism of 4 epi-lncRNAs in the malignant progression of osteosarcoma is still unknown, and whether they are cross-talk remains unclear, which needs to be further explored in future experimental studies.

In summary, our study revealed different patterns of epigenetic modification in osteosarcoma and identified epigenetically dysregulated epi-lncRNAs based on epigenetic and transcriptional analyses, which provided new insights into epigenetic regulation and identification of prognostic biomarkers in osteosarcoma.

## Data Availability Statement

Publicly available datasets were analyzed in this study. This data can be found here: transcriptome data of osteosarcoma came from TRAGET database (https://ocg.cancer.gov/programs/target). The transcriptome data of normal skeletal muscle were obtained from GTEx database (https://www.genome.gov/Funded-Programs-Projects/Genotype-Tissue-Expression-Project). The batch effect of the two datasets was eliminated by removeBatchEffect in LIMMA R package.

## Author Contributions

Conceptualization: JH and HX. Methodology, software, formal analysis, resources, data curation, writing—original draft preparation, and visualization: JH. Validation, investigation, and writing—review and editing: JZ. Supervision, project administration, and funding acquisition: HX. All authors have read and agreed to the published version of the manuscript.

## Funding

This research was funded by Shanghai Fengxian District Health Committee, Grant Number fxlczlzx-a-202103.

## Conflict of Interest

The authors declare that the research was conducted in the absence of any commercial or financial relationships that could be construed as a potential conflict of interest.

## Publisher's Note

All claims expressed in this article are solely those of the authors and do not necessarily represent those of their affiliated organizations, or those of the publisher, the editors and the reviewers. Any product that may be evaluated in this article, or claim that may be made by its manufacturer, is not guaranteed or endorsed by the publisher.

## Supplementary Material

Supplementary Figure S1 Genomic characteristics of epi-lncRNA vs. non-epi- lncRNA and epi-PCG vs. non-epi- PCG.

Supplementary Figure S2 Relationship between key lncRNA and immunity. **(A)** Correlation Heatmap between immune infiltration score calculated by three immune infiltration methods and four key lncRNAs. **(B)** Distribution differences of 28 kinds of immune infiltrating cells in patients with high and low risk groups. **(C)** Distribution differences of 10 kinds of immune infiltrating cells in patients with high and low risk groups. **(D)** Distribution difference of immune infiltration in patients with high and low risk groups. ^*^*P* < 0.05, ^**^*P* < 0.01, ^***^*P* < 0.001.

Supplementary Figure S3 Potential regulatory role of lncRNA. **(A)** Prognostic K-M curve of adjacent genes downstream of four lncRNAs. **(B)** Four lncRNAs potential Cerna networks. **(C)** Four lncRNAs potential Cerna networks were enriched into the KEGG pathway.
