# Supplementary figures and images for: Identification of Epigenetic-Dysregulated lncRNAs Signature in Osteosarcoma by Multi-Omics Data Analysis
Source: Front Med (Lausanne). 2022 Jun 16;9:892593. doi: 10.3389/fmed.2022.892593 (PMC9243510; doi:10.3389/fmed.2022.892593)

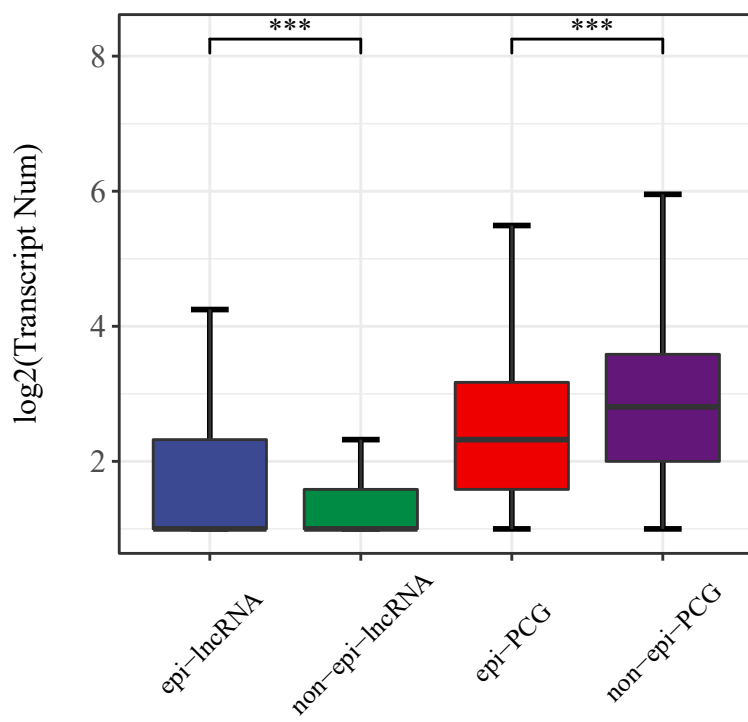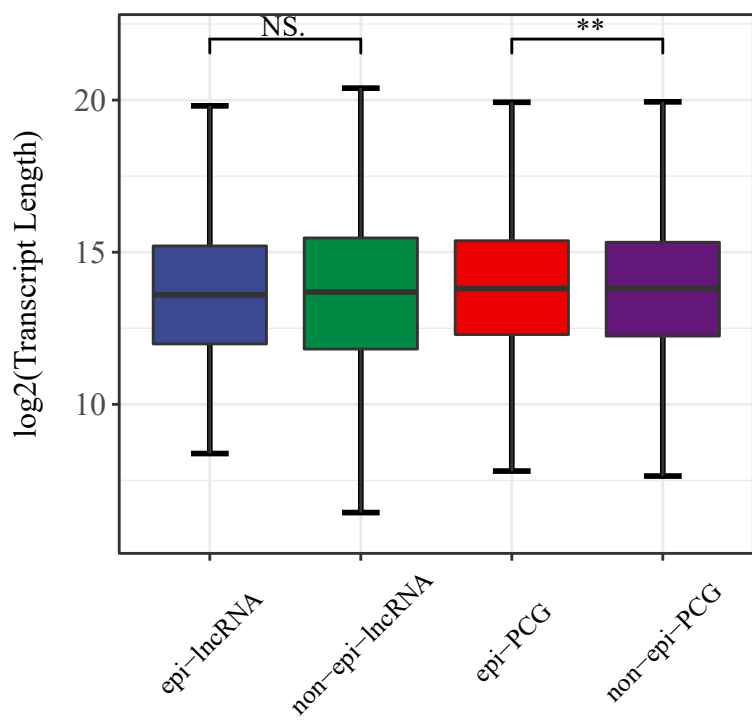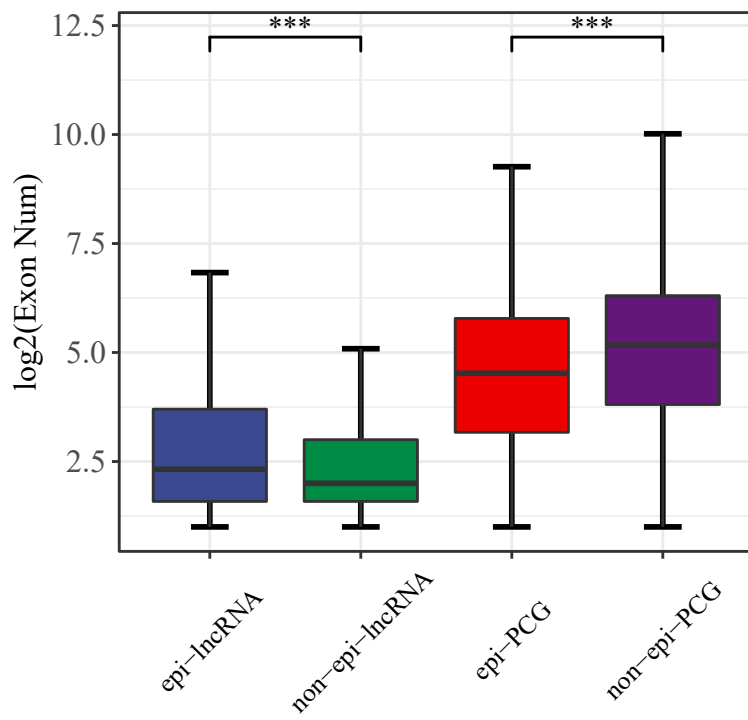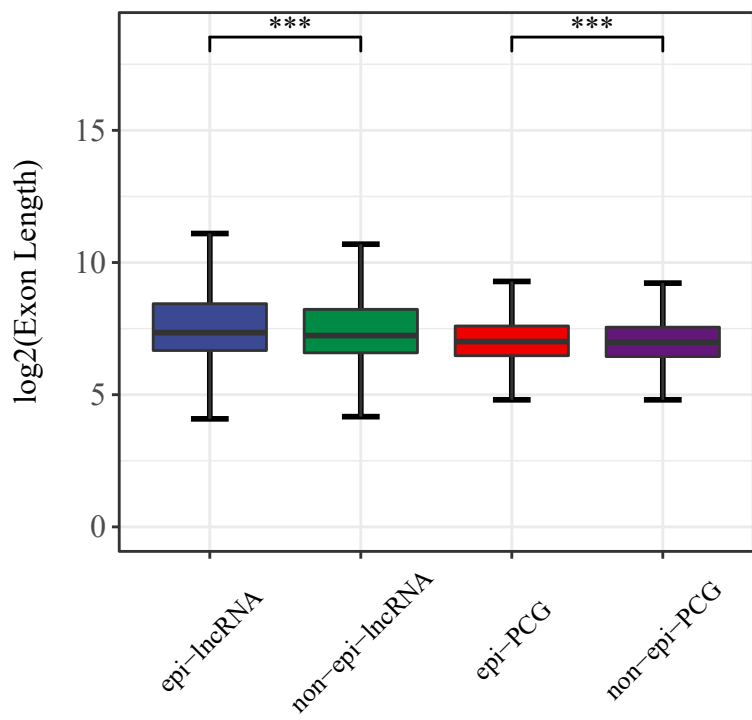

Supplement: Supplementary Figure S1 — Genomic characteristics of epi-lncRNA vs. non-epi- lncRNA and epi-PCG vs. non-epi- PCG. [file Image_1.pdf]

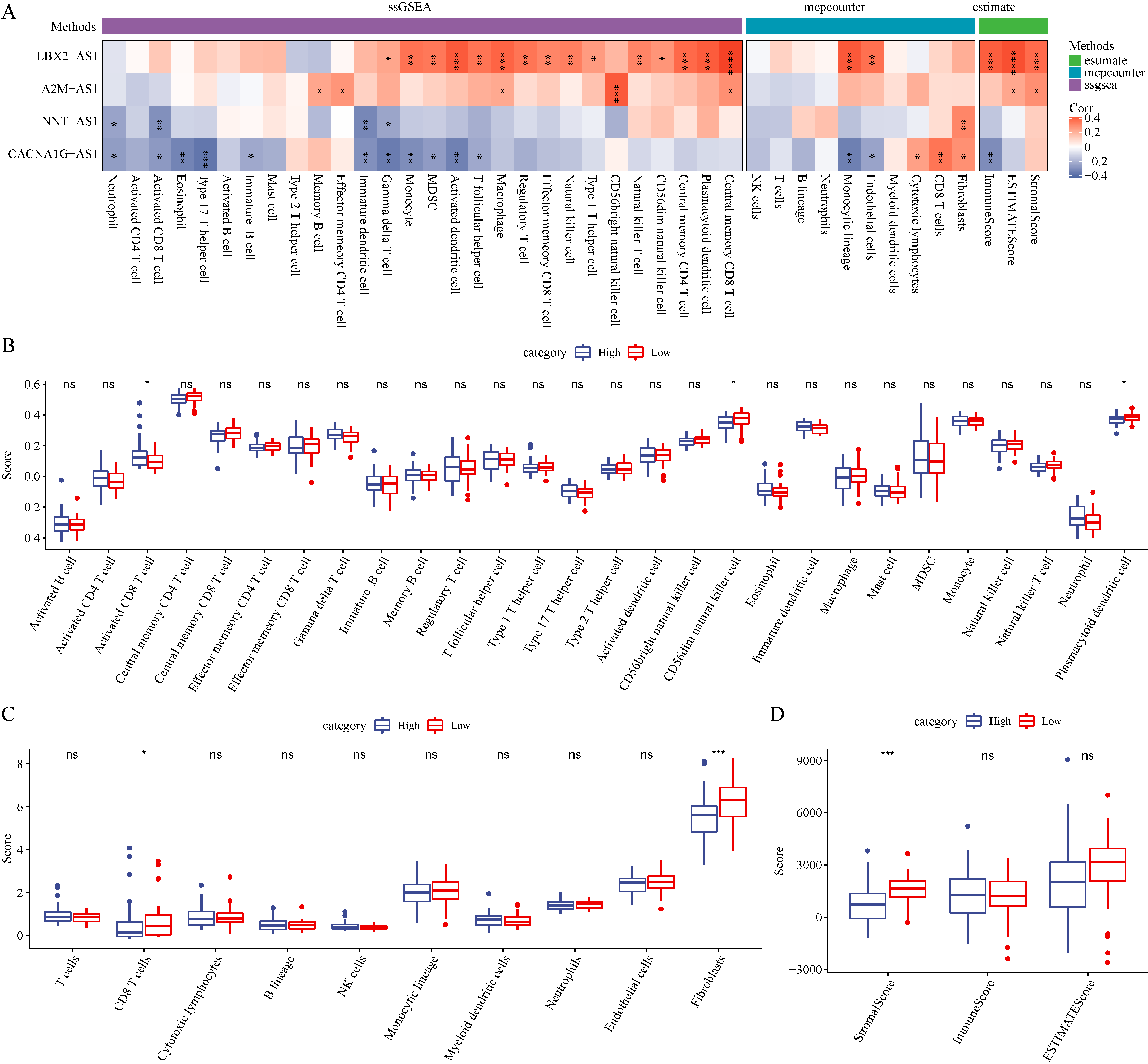

Supplement: Supplementary Figure S2 — Relationship between key lncRNA and immunity. (A) Correlation Heatmap between immune infiltration score calculated by three immune infiltration methods and four key lncRNAs. (B) Distribution differences of 28 kinds of immune infiltrating cells in patients with high and low risk groups. (C) Distribution differences of 10 kinds of immune infiltrating cells in patients with high and low risk groups. (D) Distribution difference of immune infiltration in patients with high and low risk groups. *P < 0.05, **P < 0.01, ***P < 0.001. [file Image_2.tif]

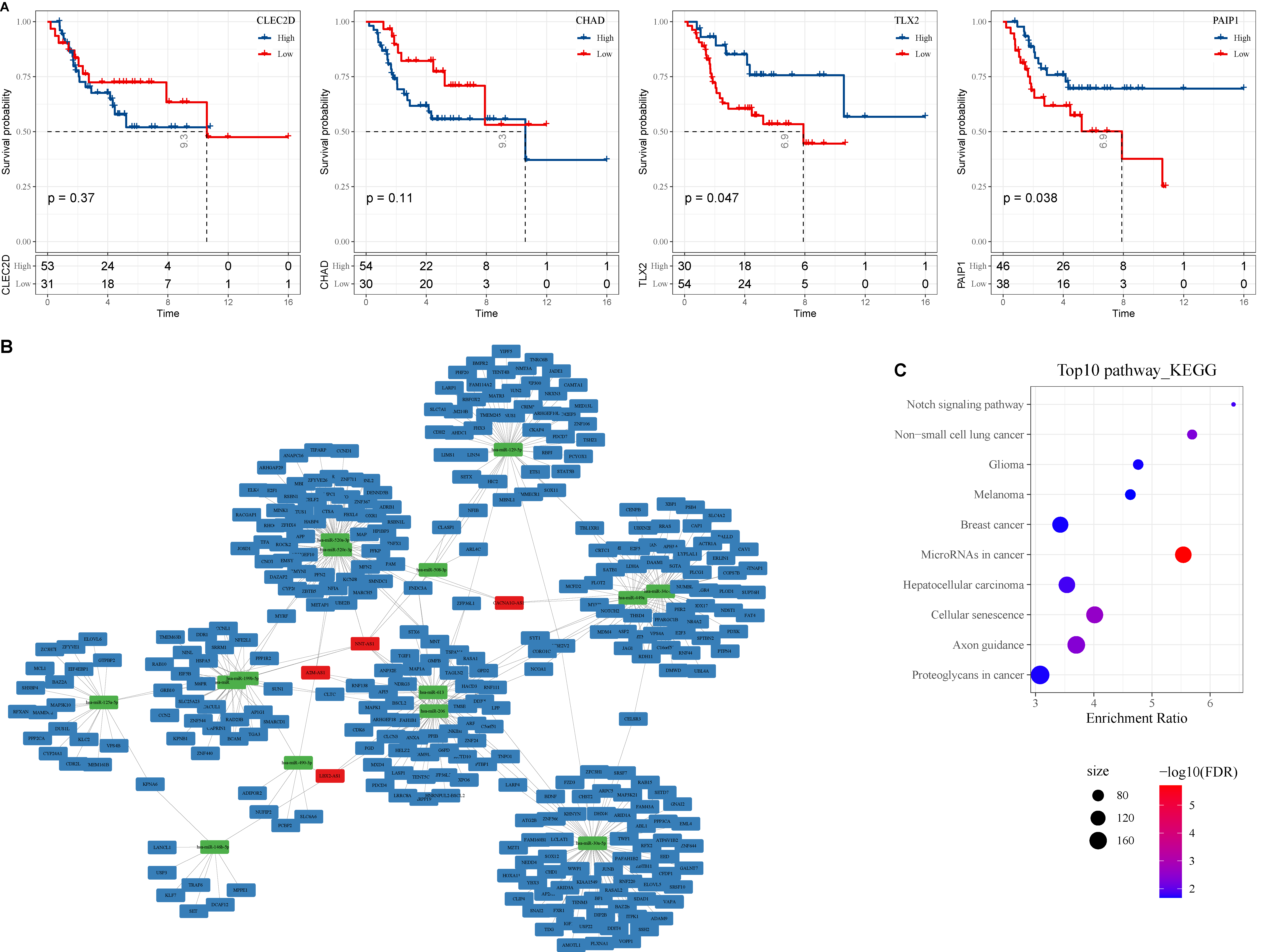

Supplement: Supplementary Figure S3 — Potential regulatory role of lncRNA. (A) Prognostic K-M curve of adjacent genes downstream of four lncRNAs. (B) Four lncRNAs potential Cerna networks. (C) Four lncRNAs potential Cerna networks were enriched into the KEGG pathway. [file Image_3.tif]
